# Supplementary material for: Distinct functions of three chromatin remodelers in activator binding and preinitiation complex assembly
Source: PLoS Genet. 2022 Jul 6;18(7):e1010277. doi: 10.1371/journal.pgen.1010277 (PMC9292117; doi:10.1371/journal.pgen.1010277)
Supplement: S1 Table — Names, genotypes and sources of each strain are given. (DOCX) [file pgen.1010277.s001.docx]

**S1 Table: Yeast strains used in this study**

| Name | Parent | Genotype | Reference |
| --- | --- | --- | --- |
| F729/BY4741 | NA | *MATa his3Δ1 leu2Δ0 met15Δ0 ura3Δ0* | Research genetics |
| F731 | BY4741 | *MATa his3Δ1 leu2Δ0 met15Δ0 ura3Δ0 gcn4∆::kanMX4* | Research genetics |
| F748 | BY4741 | *MATa his3Δ1 leu2Δ0 met15Δ0 ura3Δ0 snf2Δ::kanMX4* | Research genetics |
| HQY1632 | BY4741 | *MATa his3Δ1 leu2Δ0 met15Δ0 ura3Δ0 HIS3MX6::P_TET_STH1* | [1] |
| HQY1660 | F748 | *MATa his3Δ1 leu2Δ0 met15Δ0 ura3Δ0 snf2Δ::kanMX4 HIS3MX6::P_TET_STH1* | [1] |
| YR092 | Y24517 | *MATa his3Δ1 leu2Δ0 met15Δ0 ura3Δ0 ino80Δ::kanMX4* | [2] |
| HQY367 | BY4741 | *MATa his3Δ1 leu2Δ0 met15Δ0 ura3Δ0 SNF2-myc13::HIS3MX6* | [3] |
| HQY459 | BY4741 | *MATa his3Δ1 leu2Δ0 met15Δ0 ura3Δ0 STH1-myc13::HIS3MX6* | [3] |
| HQY1687 | BY4741 | *MATa his3Δ1 leu2Δ met15Δ ura3Δ INO80::myc13::HIS3MX6* | [2] |
| HQY1718 | F731 | *MATa his3Δ1 leu2Δ0 met15Δ0 ura3Δ0 gcn4∆::kanMX4 INO80::myc13::HIS3MX6* | This study |

*HIS3** designates the *HIS3* allele from *S. kluyveri*

**REFERENCES**

1. Rawal Y, Chereji RV, Qiu H, Ananthakrishnan S, Govind CK, Clark DJ, et al. SWI/SNF and RSC cooperate to reposition and evict promoter nucleosomes at highly expressed genes in yeast. Genes Dev. 2018;32(9-10):695-710. doi: 10.1101/gad.312850.118. PubMed PMID: 29785963; PubMed Central PMCID: PMCPMC6004078.

2. Qiu H, Biernat E, Govind CK, Rawal Y, Chereji RV, Clark DJ, et al. Chromatin remodeler Ino80C acts independently of H2A.Z to evict promoter nucleosomes and stimulate transcription of highly expressed genes in yeast. Nucleic Acids Res. 2020;48(15):8408-30. Epub 2020/07/15. doi: 10.1093/nar/gkaa571. PubMed PMID: 32663283; PubMed Central PMCID: PMCPMC7470979.

3. Swanson MJ, Qiu HF, Sumibcay L, Krueger A, Kim SJ, Natarajan K, et al. A multiplicity of coactivators is required by Gcn4p at individual promoters in vivo. Molecular and Cellular Biology. 2003;23(8):2800-20. doi: 10.1128/mcb.23.8.2800-2820.2003. PubMed PMID: WOS:000182049900015.
