# Supplementary material for: Distinct functions of three chromatin remodelers in activator binding and preinitiation complex assembly
Source: PLoS Genet. 2022 Jul 6;18(7):e1010277. doi: 10.1371/journal.pgen.1010277 (PMC9292117; doi:10.1371/journal.pgen.1010277)
Supplement: S2 Table — The strain and growth condition (SM-induced (I), or uninduced (U)), immunoprecipitating antiserum (IP), sample identification number, total number of sequencing reads (All PE reads), number of reads after removing duplicate reads (PE rmdup), correlation coefficient between indicated replicates, and source of data, are given for each ChIP-seq experiment. (DOCX) [file pgen.1010277.s002.docx]

**S2 Table: Compilation of ChIP-seq replicate experiments**

**I. Gcn4 ChIP-seq**

| Strain | IP | Sample ID | All PE reads | PE rmdup | Pearson correlation between replicates | | | Source |
| --- | --- | --- | --- | --- | --- | --- | --- | --- |
|  |  |  |  |  | AGH32_HQ46 | AGH32_HQ47 |  |  |
| WT_U | Gcn4 | AGH32  _HQ45 | 9434957 | 834910 | 0.997 | 0.997 |  | [1] |
| WT_U | Gcn4 | AGH32  _HQ46 | 8293362 | 352621 |  | 0.996 |  | [1] |
| WT_U | Gcn4 | AGH32  _HQ47 | 6312086 | 223598 |  |  |  | [1] |
|  |  |  |  |  | AGH95_10 | AGH66_01 | AGH66_02 |  |
| WT_I | Gcn4 | AGH95_09 | 23969984 | 18446498 | 0.998 | 0.973 | 0.967 | This study |
| WT_I | Gcn4 | AGH95_10 | 34793241 | 25793908 |  | 0.972 | 0.970 | This study |
| WT_I | Gcn4 | AGH66_01 | 13001067 | 3812850 |  |  | 0.992 | This study |
| WT_I | Gcn4 | AGH66_02 | 21568439 | 8750863 |  |  |  | This study |
|  |  |  |  |  | AGH95_12 |  |  |  |
| *snf2Δ*_I | Gcn4 | AGH95_11 | 16513767 | 12929335 | 0.998 |  |  | This study |
| *snf2Δ*_I | Gcn4 | AGH95_12 | 25382449 | 19395455 |  |  |  | This study |
|  |  |  |  |  | AGH96_10 | AGH66_03 | AGH66_04 |  |
| *P_TET_STH1*_I | Gcn4 | AGH96_09 | 23383351 | 17959152 | 0.990 | 0.982 | 0.980 | This study |
| *P_TET_STH1*_I | Gcn4 | AGH96_10 | 28009712 | 20396793 |  | 0.969 | 0.969 | This study |
| *P_TET_STH1*_I | Gcn4 | AGH66_03 | 13744350 | 6106482 |  |  | 0.993 | This study |
| *P_TET_STH1*_I | Gcn4 | AGH66_04 | 17305536 | 7027543 |  |  |  | This study |
|  |  |  |  |  | AGH66_05 | AGH66_06 |  |  |
| *snf2Δ P_TET_STH1*_I | Gcn4 | AGH96_11 | 35827719 | 27272344 | 0.972 | 0.972 |  | This study |
| *snf2Δ P_TET_STH1*_I | Gcn4 | AGH66_05 | 23853729 | 8885029 |  | 0.997 |  | This study |
| *snf2Δ P_TET_STH1*_I | Gcn4 | AGH66_06 | 22769050 | 9213922 |  |  |  | This study |
|  |  |  |  |  | AGH113_08 |  |  |  |
| WT_U | Gcn4 | AGH113_07 | 10542959 | 7090399 | 0.981 |  |  | This study |
| WT_U | Gcn4 | AGH113_08 | 10513497 | 6863672 |  |  |  | This study |
|  |  |  |  |  | AGH113_10 |  |  |  |
| WT_I | Gcn4 | AGH113_09 | 13698040 | 9265216 | 0.991 |  |  | This study |
| WT_I | Gcn4 | AGH113_10 | 41560943 | 28746522 |  |  |  | This study |
|  |  |  |  |  | AGH113_14 |  |  |  |
| *ino80Δ*_I | Gcn4 | AGH113_13 | 19654864 | 15014306 | 0.998 |  |  | This study |
| *ino80Δ*_I | Gcn4 | AGH113_14 | 19210671 | 14099687 |  |  |  | This study |
|  |  |  |  |  |  |  |  |  |

**S2 Table (cont’d):**

**II. TBP ChIP-seq**

| Strain | IP | Sample ID | All PE reads | PE rmdup | Pearson correlation between replicates | | Source |
| --- | --- | --- | --- | --- | --- | --- | --- |
|  |  |  |  |  | AGH121-2 | AGH121-3 | [2] |
| WT_U | TBP | AGH121-1 | 16,885,453 | 10,236,588 | 0.999 | 0.999 | [2] |
| WT_U | TBP | AGH121-2 | 27,362,033 | 12,530,926 |  | 0.999 | [2] |
| WT_U | TBP | AGH121-3 | 22,937,418 | 11,812,400 |  |  | [2] |
|  |  |  |  |  | AGH121-5 |  | [2] |
| WT_I | TBP | AGH121-4 | 24,785,155 | 14,448,990 | 0.996 |  | [2] |
| WT_I | TBP | AGH121-5 | 4,584,274 | 3,832,277 |  |  | [2] |
|  |  |  |  |  | AGH121-8 | AGH121-9 | [2] |
| *ino80Δ* _U | TBP | AGH121-7 | 26,930,283 | 18,269,275 | 0.998 | 0.999 | [2] |
| *ino80Δ* _U | TBP | AGH121-8 | 15,095,156 | 10,094,991 |  | 0.999 | [2] |
| *ino80Δ* _U | TBP | AGH121-9 | 22,489,806 | 12,887,915 |  |  | [2] |
|  |  |  |  |  | AGH121-11 | AGH121-12 | [2] |
| *ino80Δ* _I | TBP | AGH121-10 | 27,763,987 | 17,051,822 | 0.999 | 0.999 | [2] |
| *ino80Δ* _I | TBP | AGH121-11 | 27,142,493 | 18,528,771 |  | 0.999 | [2] |
| *ino80Δ* _I | TBP | AGH121-12 | 26,288,826 | 17,279,728 |  |  | [2] |
|  |  |  |  |  | AGH142_14 |  |  |
| WT_I_dox | TBP | AGH142_13 | 27411329 | 14710509 | 0.998 |  | This study |
| WT_I_dox | TBP | AGH142_14 | 31115073 | 13579121 |  |  | This study |
|  |  |  |  |  | AGH142_6 |  |  |
| *snf2Δ*_U | TBP | AGH142_5 | 23221991 | 12712910 | 0.999 |  | This study |
| *snf2Δ*_U | TBP | AGH142_6 | 29278698 | 14179344 |  |  | This study |
|  |  |  |  |  | AGH142_8 |  |  |
| *snf2Δ*_I | TBP | AGH142_7 | 26652341 | 14607284 | 0.998 |  | This study |
| *snf2Δ*_I | TBP | AGH142_8 | 25661082 | 16282120 |  |  | This study |
|  |  |  |  |  | AGH142_10 |  |  |
| *P_TET_STH1*_U_dox | TBP | AGH142_9 | 24829710 | 12561822 | 0.999 |  | This study |
| *P_TET_STH1*_U_dox | TBP | AGH142_10 | 28314060 | 12766273 |  |  | This study |
|  |  |  |  |  | AGH142_12 |  |  |
| *P_TET_STH1*_I_dox | TBP | AGH142_11 | 25442841 | 13057814 | 0.999 |  | This study |
| *P_TET_STH1*_I_dox | TBP | AGH142_12 | 25302510 | 12058412 |  |  | This study |
|  |  |  |  |  | AGH142_2 |  |  |
| *snf2∆ P_TET_STH1*_U_dox | TBP | AGH142_1 | 23,732,740 | 13,064,587 | 0.999 |  | This study |
| *snf2∆ P_TET_STH1*_U_dox | TBP | AGH142_2 | 24,739,855 | 13,218,689 |  |  | This study |
|  |  |  |  |  | AGH142_4 |  |  |
| *snf2∆ P_TET_STH1*_I_dox | TBP | AGH142_3 | 23905850 | 15330844 | 0.999 |  | This study |
| *snf2∆ P_TET_STH1*_I_dox | TBP | AGH142_4 | 28963188 | 16277896 |  |  | This study |

**S2 Table (cont’d):**

**III. CR-myc ChIP-seq**

| Strain | IP | Sample ID | All PE reads | PE rmdup | Pearson correlation between replicates | | Source |
| --- | --- | --- | --- | --- | --- | --- | --- |
|  |  |  |  |  | AGH79_10 |  |  |
| *SNF2-myc*_U | myc | AGH79_09 | 14233915 | 11112228 | 0.993 |  | This study |
| *SNF2-myc*_U | myc | AGH79_10 | 24867367 | 17476084 |  |  | This study |
|  |  |  |  |  | AGH79_12 |  |  |
| *SNF2-myc*_I | myc | AGH79_11 | 13822144 | 10588610 | 0.967 |  | This study |
| *SNF2-myc*_I | myc | AGH79_12 | 17179565 | 12644430 |  |  | This study |
|  |  |  |  |  | AGH94_10 |  |  |
| *STH1-myc*_U | myc | AGH94_09 | 17209512 | 12751019 | 0.994 |  | This study |
| *STH1-myc*_U | myc | AGH94_10 | 21946759 | 14900926 |  |  | This study |
|  |  |  |  |  | AGH94_12 |  |  |
| *STH1-myc*_I | myc | AGH94_11 | 31459556 | 21973822 | 0.948 |  | This study |
| *STH1-myc*_I | myc | AGH94_12 | 19548461 | 13093796 |  |  | This study |
|  |  |  |  |  | AGH144_2 |  |  |
| WT_U | myc | AGH144_1 | 3072942 | 2120150 | 0.992 |  | This study |
| WT_U | myc | AGH144_2 | 9738163 | 6130932 |  |  | This study |
|  |  |  |  |  | AGH144_4 |  |  |
| WT_I | myc | AGH144_3 | 11268530 | 7234492 | 0.972 |  | This study |
| WT_I | myc | AGH144_4 | 1765955 | 1301154 |  |  | This study |
|  |  |  |  |  | AGH101_18 |  |  |
| WT_I | myc | AGH101_17 | 14883149 | 12054753 | 0.991 |  | [2] |
| WT_I | myc | AGH101_18 | 11930745 | 9789686 |  |  | [2] |
|  |  |  |  |  | AGH144_6 | AGH144_7 |  |
| *INO80-myc*_U | myc | AGH144_5 | 20470589 | 14552493 | 0.990 | 0.994 | This study |
| *INO80-myc*_U | myc | AGH144_6 | 25673285 | 17246641 |  | 0.992 | This study |
| *INO80-myc*_U | myc | AGH144_7 | 19606771 | 13695074 |  |  | This study |
|  |  |  |  |  | AGH144_9 | AGH144_10 |  |
| *INO80-myc*_I | myc | AGH144_8 | 13548606 | 9478743 | 0.979 | 0.979 | This study |
| *INO80-myc*_I | myc | AGH144_9 | 21052614 | 14229783 |  | 0.991 | This study |
| *INO80-myc*_I | myc | AGH144_10 | 27494331 | 17575016 |  |  | This study |
|  |  |  |  |  | AGH144_12 |  |  |
| *gcn4∆*_U | myc | AGH144_11 | 16724373 | 10573134 | 0.996 |  | This study |
| *gcn4∆*_U | myc | AGH144_12 | 14598145 | 9133914 |  |  | This study |
|  |  |  |  |  | AGH144_14 |  |  |
| *gcn4∆*_I | myc | AGH144_13 | 20372363 | 11877487 | 0.987 |  | This study |
| *gcn4∆*_I | myc | AGH144_14 | 21985398 | 13319316 |  |  | This study |
|  |  |  |  |  | AGH144_16 | AGH144_17 |  |
| *gcn4∆ INO80-myc*_U | myc | AGH144_15 | 23070235 | 15948119 | 0.989 | 0.989 | This study |
| *gcn4∆ INO80-myc*_U | myc | AGH144_16 | 23382112 | 16195621 |  | 0.988 | This study |
| *gcn4∆ INO80-myc*_U | myc | AGH144_17 | 25118691 | 17289728 |  |  | This study |
|  |  |  |  |  | AGH144_19 | AGH144_20 |  |
| *gcn4∆ INO80-myc*_I | myc | AGH144_18 | 22775944 | 15784565 | 0.946 | 0.980 | This study |
| *gcn4∆ INO80-myc*_I | myc | AGH144_19 | 29498726 | 18524887 |  | 0.982 | This study |
| *gcn4∆ INO80-myc*_I | myc | AGH144_20 | 22367225 | 15448406 |  |  | This study |

**S2 Table (cont’d):**

**IV. Rpb3 ChIP-seq**

| Strain | IP | Sample ID | All PE reads | PE rmdup | Pearson correlation between replicates | | | Source |
| --- | --- | --- | --- | --- | --- | --- | --- | --- |
|  |  |  |  |  | AGH03_2 | AGH03_3 |  |  |
| WT_U | Rpb3 | AGH03_1 | 12,103,981 | 7,624,104 | 0.997 | 0.997 |  | [3] |
| WT_U | Rpb3 | AGH03_2 | 15,621,065 | 10,271,685 |  | 0.994 |  | [3] |
| WT_U | Rpb3 | AGH03_3 | 14,624,526 | 8,895,936 |  |  |  | [3] |
|  |  |  |  |  | AGH101_10 |  |  |  |
| *snf2Δ* _U | Rpb3 | AGH101_09 | 18890173 | 15825662 | 0.999 |  |  | This study |
| *snf2Δ* _U | Rpb3 | AGH101_10 | 19845532 | 15822921 |  |  |  | This study |
|  |  |  |  |  | AGH101_12 |  |  |  |
| *P_TET_STH1*_U | Rpb3 | AGH101_11 | 16038655 | 13091284 | 0.999 |  |  | This study |
| *P_TET_STH1*_U | Rpb3 | AGH101_12 | 16877236 | 13736217 |  |  |  | This study |
|  |  |  |  |  | AGH101_14 |  |  |  |
| *snf2Δ P_TET_STH1*_U | Rpb3 | AGH101_13 | 18258099 | 15112166 | 0.998 |  |  | This study |
| *snf2Δ P_TET_STH1*_U | Rpb3 | AGH101_14 | 23241654 | 18587864 |  |  |  | This study |
|  |  |  |  |  | AGH64_8 | AGH64_9 |  |  |
| *ino80Δ* _U | Rpb3 | AGH64_7 | 6602236 | 3364700 | 0.993 | 0.993 |  | This study |
| *ino80Δ* _U | Rpb3 | AGH64_8 | 7066940 | 3954861 |  | 0.995 |  | This study |
| *ino80Δ* _U | Rpb3 | AGH64_9 | 8155385 | 3999284 |  |  |  | This study |
|  |  |  |  |  | AGH03_5 | AGH03_6 |  |  |
| WT_I | Rpb3 | AGH03_4 | 10,978,154 | 7,859,118 | 0.996 | 0.996 |  | [3] |
| WT_I | Rpb3 | AGH03_5 | 16,862,702 | 11,526,037 |  | 0.995 |  | [3] |
| WT_I | Rpb3 | AGH03_6 | 14,660,604 | 10,042,576 |  |  |  | [3] |
|  |  |  |  |  | AGH12_8 | AGH28_1 |  |  |
| *snf2Δ* _I | Rpb3 | AGH12_7 | 14213867 | 10131580 | 0.995 | 0.938 |  | [3] |
| *snf2Δ* _I | Rpb3 | AGH12_8 | 12857958 | 8200570 |  | 0.950 |  | [3] |
| *snf2Δ* _I | Rpb3 | AGH28_1 | 13651250 | 1859651 |  |  |  | [3] |
|  |  |  |  |  | AGH51_05 | AGH51_06 |  |  |
| *P_TET_STH1*_I | Rpb3 | AGH51_01 | 6836169 | 1768416 | 0.993 | 0.990 |  | [4] |
| *P_TET_STH1*_I | Rpb3 | AGH51_05 | 12990191 | 2395951 |  | 0.992 |  | [4] |
| *P_TET_STH1*_I | Rpb3 | AGH51_06 | 13376423 | 2103356 |  |  |  | [4] |
|  |  |  |  |  | AGH51_09 | AGH51_10 |  |  |
| *snf2Δ P_TET_STH1*_I | Rpb3 | AGH51_02 | 12853198 | 1909934 | 0.987 | 0.986 |  | [4] |
| *snf2Δ P_TET_STH1*_I | Rpb3 | AGH51_09 | 11798395 | 1996785 |  | 0.990 |  | [4] |
| *snf2Δ P_TET_STH1*_I | Rpb3 | AGH51_10 | 12653664 | 2171622 |  |  |  | [4] |
|  |  |  |  |  | AGH64-11 | AGH64-12 |  |  |
| *ino80Δ* _I | Rpb3 | AGH64-10 | 10,776,203 | 5,019,827 | 0.9943 | 0.9937 |  | [2] |
| *ino80Δ* _I | Rpb3 | AGH64-11 | 8,847,337 | 4,762,328 |  | 0.9956 |  | [2] |
| *ino80Δ* _I | Rpb3 | AGH64-12 | 8,315,372 | 3,731,210 |  |  |  | [2] |

**S2 Table (cont’d):**

**V. Sonication H3 (SC_H3) ChIP-seq**

| Strain | IP | Sample ID | All PE reads | PE rmdup | Pearson correlation between replicates | | | | | Source |
| --- | --- | --- | --- | --- | --- | --- | --- | --- | --- | --- |
|  |  |  |  |  | AGH0220-2 | AGH0220-3 | AGH58_02 | AGH62_01 | AGH62_02 |  |
| WT_U | SC_H3 | AGH0220-1 | 12,305,230 | 11,468,229 | 0.965 | 0.967 | 0.850 | 0.907 | 0.911 | [3] |
| WT_U | SC_H3 | AGH0220-2 | 11,725,253 | 10,818,529 |  | 0.964 | 0.850 | 0.904 | 0.904 | [3] |
| WT_U | SC_H3 | AGH0220-3 | 12,511,668 | 11,629,718 |  |  | 0.868 | 0.916 | 0.915 | [3] |
| WT_U | SC_H3 | AGH58_02 | 29625671 | 8011769 |  |  |  | 0.923 | 0.908 | [4] |
| WT_U | SC_H3 | AGH62_01 | 14993257 | 8597216 |  |  |  |  | 0.966 | [4] |
| WT_U | SC_H3 | AGH62_02 | 19032734 | 9965205 |  |  |  |  |  | [4] |
|  |  |  |  |  | AGH0220-5 | AGH0220-6 | AGH58-04 | AGH62_03 | AGH62_08 |  |
| WT_I | SC_H3 | AGH0220-4 | 9,947,964 | 7,013,803 | 0.947 | 0.939 | 0.903 | 0.937 | 0.915 | [3] |
| WT_I | SC_H3 | AGH0220-5 | 10,810,351 | 10,096,814 |  | 0.968 | 0.872 | 0.930 | 0.901 | [3] |
| WT_I | SC_H3 | AGH0220-6 | 11,687,103 | 10,849,724 |  |  | 0.850 | 0.916 | 0.884 | [3] |
| WT_I | SC_H3 | AGH58-04 | 20,620,578 | 5,466,934 |  |  |  | 0.936 | 0.933 | [4] |
| WT_I | SC_H3 | AGH62-03 | 18,617,080 | 8,200,524 |  |  |  |  | 0.951 | [4] |
| WT_I | SC_H3 | AGH62-08 | 19,798,491 | 5,044,590 |  |  |  |  |  | [4] |
|  |  |  |  |  | AGH04_02 | AGH25_01 | AGH59_01 | AGH59_02 | AGH62_05 |  |
| *snf2Δ* _I | SC_H3 | AGH04_01 | 15597956 | 11985536 | 0.963 | 0.881 | 0.905 | 0.897 | 0.870 | [3] |
| *snf2Δ* _I | SC_H3 | AGH04_02 | 17071372 | 13099928 |  | 0.871 | 0.916 | 0.907 | 0.883 | [3] |
| *snf2Δ* _I | SC_H3 | AGH25_01 | 24839200 | 11548218 |  |  | 0.796 | 0.799 | 0.720 | [3] |
| *snf2Δ* _I | SC_H3 | AGH59_01 | 14174694 | 7052399 |  |  |  | 0.951 | 0.947 | [4] |
| *snf2Δ* _I | SC_H3 | AGH59_02 | 14573693 | 6456125 |  |  |  |  | 0.945 | [4] |
| *snf2Δ* _I | SC_H3 | AGH62_05 | 20877009 | 10613818 |  |  |  |  |  | [4] |
|  |  |  |  |  | AGH50_06 | AGH54_03 |  |  |  |  |
| *P_TET_STH1*_I | SC_H3 | AGH50_01 | 23176098 | 12629620 | 0.962 | 0.970 |  |  |  | [4] |
| *P_TET_STH1*_I | SC_H3 | AGH50_06 | 26443231 | 6284621 |  | 0.971 |  |  |  | [4] |
| *P_TET_STH1*_I | SC_H3 | AGH54_03 | 22858502 | 9011007 |  |  |  |  |  | [4] |
|  |  |  |  |  | AGH54_01 | AGH54_02 |  |  |  |  |
| *snf2Δ P_TET_STH1*_I | SC_H3 | AGH50_10 | 30027139 | 5408247 | 0.863 | 0.881 |  |  |  | [4] |
| *snf2Δ P_TET_STH1*_I | SC_H3 | AGH54_01 | 23674356 | 12035478 |  | 0.951 |  |  |  | [4] |
| *snf2Δ P_TET_STH1*_I | SC_H3 | AGH54_02 | 27937517 | 13179773 |  |  |  |  |  | [4] |
|  |  |  |  |  | AGH61-5 | AGH61-6 |  |  |  |  |
| *ino80Δ* _I | SC_H3 | AGH61-4 | 16,858,717 | 8,430,284 | 0.942 | 0.942 |  |  |  | [2] |
| *ino80Δ* _I | SC_H3 | AGH61-5 | 18,996,243 | 9,997,136 |  | 0.947 |  |  |  | [2] |
| *ino80Δ* _I | SC_H3 | AGH61-6 | 19,488,986 | 10,065,366 |  |  |  |  |  | [2] |

**S2 Table (cont’d):**

**VI. MNase H3 (MN_H3) ChIP-seq**

| Strain | IP | Sample ID | All PE reads | PE rmdup | Pearson correlation between replicates | | | | | Source |
| --- | --- | --- | --- | --- | --- | --- | --- | --- | --- | --- |
|  |  |  |  |  | AGH68_02 | AGH73_01 | AGH73_02 |  |  |  |
| WT_U | MN_H3 | AGH68_01 | 21155789 | 16387173 | 0.975 | 0.939 | 0.971 |  |  | [4] |
| WT_U | MN_H3 | AGH68_02 | 29890860 | 21290074 |  | 0.941 | 0.975 |  |  | [4] |
| WT_U | MN_H3 | AGH73_01 | 22748400 | 13752475 |  |  | 0.956 |  |  | [4] |
| WT_U | MN_H3 | AGH73_02 | 33931620 | 20843860 |  |  |  |  |  | [4] |
|  |  |  |  |  | AGH68_04 | AGH73_03 |  |  |  |  |
| WT_I | MN_H3 | AGH68_03 | 22985503 | 17204691 | 0.957 | 0.926 |  |  |  | [4] |
| WT_I | MN_H3 | AGH68_04 | 19959153 | 15376225 |  | 0.956 |  |  |  | [4] |
| WT_I | MN_H3 | AGH73_03 | 26493831 | 17942518 |  |  |  |  |  | [4] |
|  |  |  |  |  | AGH68_06 | AGH73_04 | AGH73_05 | AGH73_10 |  |  |
| *snf2Δ* _I | MN_H3 | AGH68_05 | 20896164 | 15789365 | 0.964 | 0.961 | 0.966 | 0.949 |  | [4] |
| *snf2Δ* _I | MN_H3 | AGH68_06 | 23587865 | 18055776 |  | 0.972 | 0.965 | 0.965 |  | [4] |
| *snf2Δ* _I | MN_H3 | AGH73_04 | 30193725 | 20155496 |  |  | 0.977 | 0.972 |  | [4] |
| *snf2Δ* _I | MN_H3 | AGH73_05 | 28834376 | 19622426 |  |  |  | 0.964 |  | [4] |
| *snf2Δ* _I | MN_H3 | AGH73_10 | 27351420 | 18319655 |  |  |  |  |  | [4] |
|  |  |  |  |  | AGH68_08 | AGH73_06 | AGH73_07 | AGH73_12 |  |  |
| *P_TET_STH1*_I | MN_H3 | AGH68_07 | 21614150 | 16939365 | 0.980 | 0.974 | 0.960 | 0.933 |  | [4] |
| *P_TET_STH1*_I | MN_H3 | AGH68_08 | 24081014 | 18755148 |  | 0.977 | 0.964 | 0.930 |  | [4] |
| *P_TET_STH1*_I | MN_H3 | AGH73_06 | 29619041 | 19719008 |  |  | 0.979 | 0.958 |  | [4] |
| *P_TET_STH1*_I | MN_H3 | AGH73_07 | 26053659 | 18067975 |  |  |  | 0.971 |  | [4] |
| *P_TET_STH1*_I | MN_H3 | AGH73_12 | 24618600 | 16927985 |  |  |  |  |  | [4] |
|  |  |  |  |  | AGH68_10 | AGH68_11 | AGH68_12 | AGH73_08 | AGH73_09 |  |
| *snf2Δ P_TET_STH1*_I | MN_H3 | AGH68_09 | 24352916 | 18823892 | 0.961 | 0.975 | 0.911 | 0.968 | 0.967 | [4] |
| *snf2Δ P_TET_STH1*_I | MN_H3 | AGH68_10 | 35625027 | 24578682 |  | 0.949 | 0.897 | 0.947 | 0.943 | [4] |
| *snf2Δ P_TET_STH1*_I | MN_H3 | AGH68_11 | 26104427 | 19584798 |  |  | 0.913 | 0.966 | 0.969 | [4] |
| *snf2Δ P_TET_STH1*_I | MN_H3 | AGH68_12 | 19106755 | 11875757 |  |  |  | 0.883 | 0.891 | [4] |
| *snf2Δ P_TET_STH1*_I | MN_H3 | AGH73_08 | 27010389 | 18495503 |  |  |  |  | 0.976 | [4] |
| *snf2Δ P_TET_STH1*_I | MN_H3 | AGH73_09 | 25011131 | 17657515 |  |  |  |  |  | [4] |

**REFERENCES**

1. Rawal Y, Chereji RV, Valabhoju V, Qiu H, Ocampo J, Clark DJ, et al. Gcn4 Binding in Coding Regions Can Activate Internal and Canonical 5' Promoters in Yeast. Mol Cell. 2018;70(2):297-311.e4. Epub 2018/04/05. doi: 10.1016/j.molcel.2018.03.007. PubMed PMID: 29628310; PubMed Central PMCID: PMCPMC6133248.

2. Qiu H, Biernat E, Govind CK, Rawal Y, Chereji RV, Clark DJ, et al. Chromatin remodeler Ino80C acts independently of H2A.Z to evict promoter nucleosomes and stimulate transcription of highly expressed genes in yeast. Nucleic Acids Res. 2020;48(15):8408-30. Epub 2020/07/15. doi: 10.1093/nar/gkaa571. PubMed PMID: 32663283; PubMed Central PMCID: PMCPMC7470979.

3. Qiu H, Chereji RV, Hu C, Cole HA, Rawal Y, Clark DJ, et al. Genome-wide cooperation by HAT Gcn5, remodeler SWI/SNF, and chaperone Ydj1 in promoter nucleosome eviction and transcriptional activation. Genome Res. 2016;26(2):211-25. doi: 10.1101/gr.196337.115. PubMed PMID: 26602697; PubMed Central PMCID: PMCPMC4728374.

4. Rawal Y, Chereji RV, Qiu H, Ananthakrishnan S, Govind CK, Clark DJ, et al. SWI/SNF and RSC cooperate to reposition and evict promoter nucleosomes at highly expressed genes in yeast. Genes Dev. 2018;32(9-10):695-710. doi: 10.1101/gad.312850.118. PubMed PMID: 29785963; PubMed Central PMCID: PMCPMC6004078.
