# Supplementary material for: Distinct functions of three chromatin remodelers in activator binding and preinitiation complex assembly
Source: PLoS Genet. 2022 Jul 6;18(7):e1010277. doi: 10.1371/journal.pgen.1010277 (PMC9292117; doi:10.1371/journal.pgen.1010277)
Supplement: S3 Table — Mean occupancies (+/- SEM or +/- SD) of Gcn4 in the UAS regions of the indicated genes is expressed as the ratio of input DNA recovered in the immunoprecipitates corrected for the same ratio measured for non-transcribed sequences from chromosome V (analyzed as a control for non-specific immunoprecipitation) as described previously [30]. (DOCX) [file pgen.1010277.s003.docx]

# S3 Table. PCR ChIP data for Gcn4 binding at four exemplar target genes.

1. **Gcn4 binding at promoter (data from Fig 1D of Qiu *et al.* 2016)**

|  | Average | | | | SE | | | |
| --- | --- | --- | --- | --- | --- | --- | --- | --- |
|  | *ARG1* | *ARG4* | *HIS4* | *CPA2* | *ARG1* | *ARG4* | *HIS4* | *CPA2* |
| WT_I | 30.8 | 22.7 | 21.8 | 33.6 | 4.5 | 3.3 | 1.8 | 2.2 |
| *snf2∆_*I | 63.2 | 37.4 | 21.7 | 51.5 | 7.5 | 4.2 | 1.4 | 2.4 |

1. **Gcn4 binding at promoter**

|  | Average | | | | SD | | | |
| --- | --- | --- | --- | --- | --- | --- | --- | --- |
|  | *ARG1* | *ARG4* | *HIS4* | *CPA2* | *ARG1* | *ARG4* | *HIS4* | *CPA2* |
| WT_I | 5.62 | 4.75 | 4.45 | 4.46 | 0.67 | 0.44 | 1.37 | 0.05 |
| *P_TET_-STH1_*I | 5.94 | 3.79 | 4.12 | 4.02 | 0.31 | 0.41 | 0.13 | 0.36 |
| *snf2∆ P_TET_-STH1_*I | 6.73 | 3.50 | 2.75 | 3.91 | 1.39 | 0.01 | 0.01 | 0.79 |

# Mean occupancies (+/- SEM or +/- SD) of Gcn4 in the UAS regions of the indicated genes is expressed as the ratio of input DNA recovered in the immunoprecipitates corrected for the same ratio measured for non-transcribed sequences from chromosome V (analyzed as a control for non-specific immunoprecipitation) as described previously [1].

**REFERENCE**

1. Qiu H, Chereji RV, Hu C, Cole HA, Rawal Y, Clark DJ, et al. Genome-wide cooperation by HAT Gcn5, remodeler SWI/SNF, and chaperone Ydj1 in promoter nucleosome eviction and transcriptional activation. Genome Res. 2015. doi: 10.1101/gr.196337.115. PubMed PMID: 26602697.
