# Supplementary material for: Distinct functions of three chromatin remodelers in activator binding and preinitiation complex assembly
Source: PLoS Genet. 2022 Jul 6;18(7):e1010277. doi: 10.1371/journal.pgen.1010277 (PMC9292117; doi:10.1371/journal.pgen.1010277)
Supplement: S1 Fig — (A-B) Box plots of log2 Gcn4 occupancies in biological replicates of WT_U, WT_I, or snf2Δ_I, PTET-STH1_I and snf2Δ PTET-STH1_I cells for all 5’ sites (A), or in the three sets of Gcn4 5’ sites defined in Fig 2B (B). (C) Box plots of H3 occupancies in biological replicates of WT_U, WT_I, or snf2Δ_I, PTET-STH1_I and snf2Δ PTET-STH1_I cells for the three sets of 5’ Gcn4 sites defined in Fig 2B. (DOCX) [file pgen.1010277.s004.docx]

**S1 Fig. Supporting evidence that SWI/SNF and RSC have differential effects on Gcn4 binding at 5’ sites. (A-B)** Box plots of log_2_ Gcn4 occupancies in biological replicates of WT_U, WT_I, or *snf2Δ*_I, *P_TET_-STH1_*I and *snf2Δ P_TET_-STH1_*I cells for all 5’ sites (A), or in the three sets of Gcn4 5’ sites defined in Fig 2B (B). **(C)** Box plots of H3 occupancies in biological replicates of WT_U, WT_I, or *snf2Δ*_I, *P_TET_-STH1_*I and *snf2Δ P_TET_-STH1_*I cells for the three sets of 5’ Gcn4 sites defined in Fig 2B.
