# Supplementary material for: Distinct functions of three chromatin remodelers in activator binding and preinitiation complex assembly
Source: PLoS Genet. 2022 Jul 6;18(7):e1010277. doi: 10.1371/journal.pgen.1010277 (PMC9292117; doi:10.1371/journal.pgen.1010277)
Supplement: S3 Fig — (A-C) Paired box plots of log2 Gcn4 occupancies comparing (A) WT_I versus PTET-STH1_I, (B) PTET-STH1_I versus WT_I and snf2Δ PTET-STH1_I and (C) WT_I versus snf2Δ_I in 3 sets of Gcn4 5’ sites comprised of the (i) first (Set_1, n = 30), (ii) middle two (Set_2, n = 57) and (iii) last (Set_3, n = 30) quartiles of the fold-changes in Gcn4 occupancy in snf2Δ PTET-STH1_I vs. WT_I cells as depicted in Fig 2B(i). Lines connecting each data point in respective strains indicate changes in the Gcn4 occupancies of respective Gcn4 site. (D) Sectored scatterplot of the log2 ratios of Gcn4 occupancies per base pair over the peak coordinates assigned by MACS2 analysis in snf2Δ_I vs. WT_I cells versus the corresponding log2 ratios of Gcn4 occupancies in snf2Δ PTET-STH1_I vs. WT_I cells. The 3 sets of Gcn4 5’ sites defined in Fig 2B(ii) are color-coded as: Set_1, red rectangles; Set_2, green pluses; and Set_3, blue stars. (DOCX) [file pgen.1010277.s006.docx]

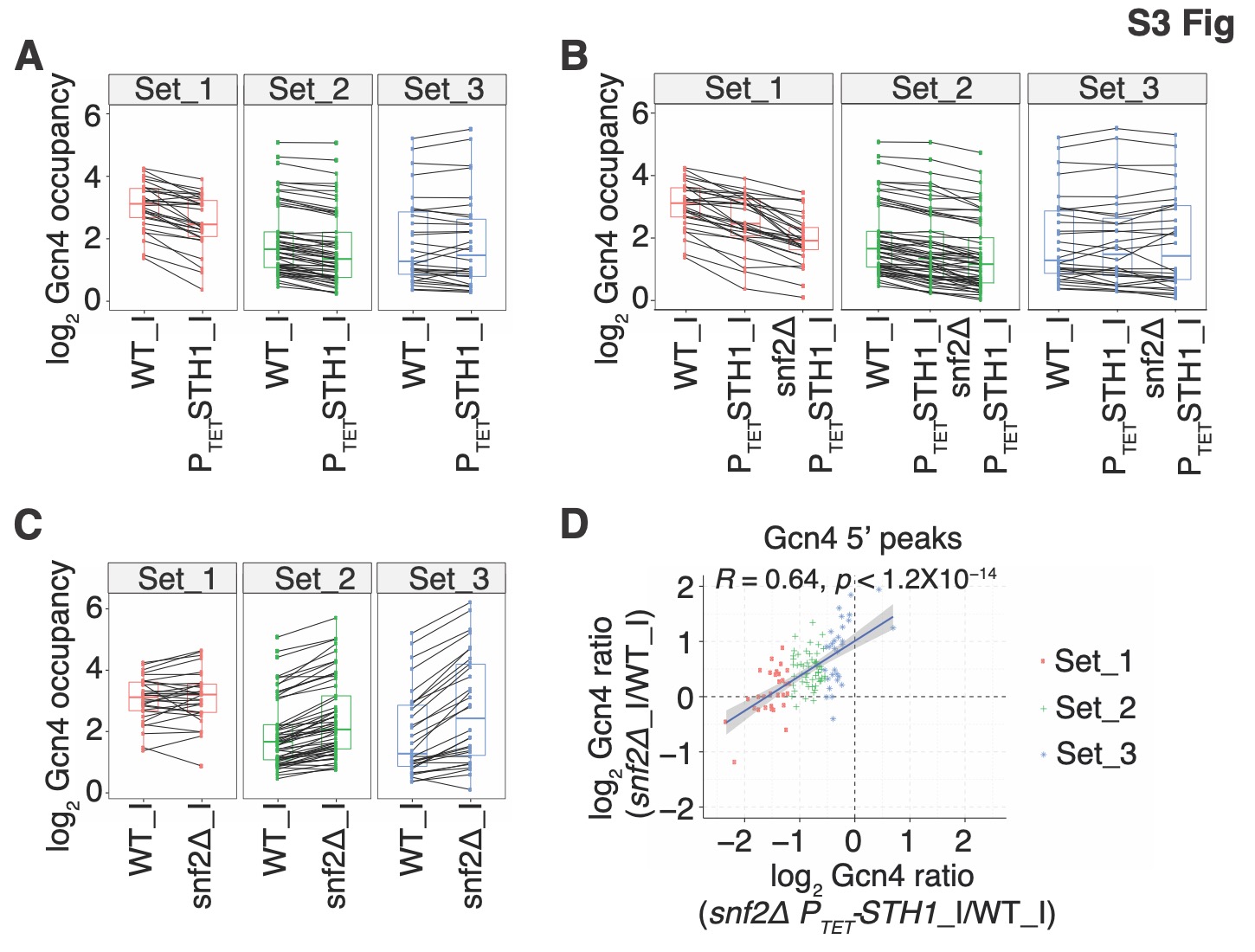


**S3 Fig. Supporting evidence that SWI/SNF and RSC have differential effects on Gcn4 binding at 5’ sites. (A-C)** Paired box plots of log_2_ Gcn4 occupancies comparing (A) WT_I versus *P_TET_-STH1_*I, (B) *P_TET_-STH1_*I versus WT_I and *snf2Δ P_TET_-STH1_*I and (C) WT_I versus *snf2Δ*_I in 3 sets of Gcn4 5’ sites comprised of the (i) first (Set_1, n=30), (ii) middle two (Set_2, n=57) and (iii) last (Set_3, n=30) quartiles of the fold-changes in Gcn4 occupancy in *snf2Δ P_TET_-STH1_*I vs. WT_I cells as depicted in Fig 2B(i). Lines connecting each data point in respective strains indicate changes in the Gcn4 occupancies of respective Gcn4 site. **(D)** Sectored scatterplot of the log_2_ ratios of Gcn4 occupancies per base pair over the peak coordinates assigned by MACS2 analysis in *snf2Δ_*I vs. WT_I cells versus the corresponding log_2_ ratios of Gcn4 occupancies in *snf2Δ P_TET_-STH1_*I vs. WT_I cells*.*  The 3 sets of Gcn4 5’ sites defined in Fig 2B(ii) are color-coded as: Set_1, red rectangles; Set_2, green pluses; and Set_3, blue stars.
