# Supplementary material for: Distinct functions of three chromatin remodelers in activator binding and preinitiation complex assembly
Source: PLoS Genet. 2022 Jul 6;18(7):e1010277. doi: 10.1371/journal.pgen.1010277 (PMC9292117; doi:10.1371/journal.pgen.1010277)
Supplement: S4 Fig — The Gcn4 peak numbering assigned previously [46] is given at the top of each profile, and the Gcn4 occupancies per nucleotide averaged over the peaks determined in this study are listed next to each peak. (DOCX) [file pgen.1010277.s007.docx]

**S4 Fig. Gene browser profiles of Gcn4 and H3 occupancies from ChIP-seq analyses of sonicated chromatin for representative genes in the indicated strains**. The Gcn4 peak numbering assigned previously **[1]** is given at the top of each profile, and the Gcn4 occupancies per nucleotide averaged over the peaks determined in this study are listed next to each peak.

**REFERENCE**

1. Rawal Y, Chereji RV, Valabhoju V, Qiu H, Ocampo J, Clark DJ, et al. Gcn4 Binding in Coding Regions Can Activate Internal and Canonical 5' Promoters in Yeast. Mol Cell. 2018;70(2):297-311 e4. doi: 10.1016/j.molcel.2018.03.007. PubMed PMID: 29628310.
