# Supplementary material for: Distinct functions of three chromatin remodelers in activator binding and preinitiation complex assembly
Source: PLoS Genet. 2022 Jul 6;18(7):e1010277. doi: 10.1371/journal.pgen.1010277 (PMC9292117; doi:10.1371/journal.pgen.1010277)
Supplement: S5 Fig — (A) Heat maps of differences in Gcn4 occupancies averaged across the coordinates of 5’ sites between the indicated mutant and WT_I samples for (i) ino80Δ_I and (ii) snf2Δ PTET-STH1_I. Gcn4 5’ sites were sorted by increasing order of the ratio of Gcn4 occupancies in the double mutant snf2Δ PTET-STH1_I vs. WT_I. (B) Heat map depictions of Gcn4 occupancies surrounding the Gcn4 motifs of 5’ sites in (i) WT_I (for the ino80Δ mutant), (ii) ino80Δ_I, (iii) WT_I (for the snf2Δ PTET-STH1_I mutant), and (iv) snf2Δ PTET-STH1_I cells, for the same ordering of 5’ sites as in (A). The sets of Gcn4 5’ sites (Set_1, Set_2, and Set_3) defined in Fig 2A and 2B are depicted in B(i). (C & D) Same analyses for ino80Δ_I and PTET-STH1_I as shown in (A-B) except for Gcn4 ORF sites. (DOCX) [file pgen.1010277.s008.docx]

**S5 Fig. Differential requirements for Ino80C and RSC for Gcn4 binding at a subset of Gcn4 5’ sites.** (A) Heat maps of differences in Gcn4 occupancies averaged across the coordinates of 5’ sites between the indicated mutant and WT_I samples for (i) *ino80∆*_I and (ii) *snf2Δ P_TET_-STH1*_I. Gcn4 5’ sites were sorted by increasing order of the ratio of Gcn4 occupancies in the double mutant *snf2Δ P_TET_-STH1*_I vs. WT_I. (B) Heat map depictions of Gcn4 occupancies surrounding the Gcn4 motifs of 5’ sites in (i) WT_I (for the *ino80Δ* mutant), (ii) *ino80∆*_I, (iii) WT_I (for the *snf2Δ* *P_TET_-STH1*_I mutant), and (iv) *snf2Δ* *P_TET_-STH1*_I cells, for the same ordering of 5’ sites as in (A). The sets of Gcn4 5’ sites (Set_1, Set_2, and Set_3) defined in Fig 2A-B are depicted in B(i). (C & D) Same analyses for *ino80∆*_I and *P_TET_-STH1*_I as shown in (A-B) except for Gcn4 ORF sites.
