# Supplementary material for: Distinct functions of three chromatin remodelers in activator binding and preinitiation complex assembly
Source: PLoS Genet. 2022 Jul 6;18(7):e1010277. doi: 10.1371/journal.pgen.1010277 (PMC9292117; doi:10.1371/journal.pgen.1010277)
Supplement: S6 Fig — (A) Heat map depicting differences in Gcn4 occupancies between ino80Δ_I and WT_I cells (i); Gcn4 occupancies surrounding the motifs of 5’ sites in (ii) WT_I or (iii) ino80Δ_I cells. Gcn4 5’ sites were sorted by increasing order of the ratio of Gcn4 occupancies in ino80Δ_I vs. WT_I cells, and the first (Set_I, n = 30), middle two (Set_II, n = 57) and fourth (Set_III, n = 30) quartiles of fold-changes are depicted in A(ii). (B) Heat map depictions of differences in Gcn4 occupancies between (i) snf2Δ PTET-STH1_I and WT_I, (ii) PTET-STH1_I and WT_I, and (iii) snf2_I vs. WT_I cells in same order as in S6A Fig. (C) Notched box plots of log2 Gcn4 occupancy in WT_U, WT_I, and ino80Δ_I cells in 3 sets of Gcn4 5’ sites comprised of the (i) first (Set_I, n = 30), (ii) middle two (Set_II, n = 57) and (iii) last (Set_III, n = 30) quartiles of the fold-changes in Gcn4 occupancy in ino80Δ_I vs. WT_I cells as defined in panel A(ii). P values for the significance of differences in medians calculated by the Mann-Whitney-Wilcoxon test are indicated. (D) Scatterplots of log2 ratios of Gcn4 occupancy changes in WT_I vs. ino80Δ_I cells plotted against log2 Gcn4 occupancies in WT_I cells for 5’ (i) and ORF (ii) Gcn4 sites. Pearson correlation coefficients (R) and associated p values are indicated. (DOCX) [file pgen.1010277.s009.docx]

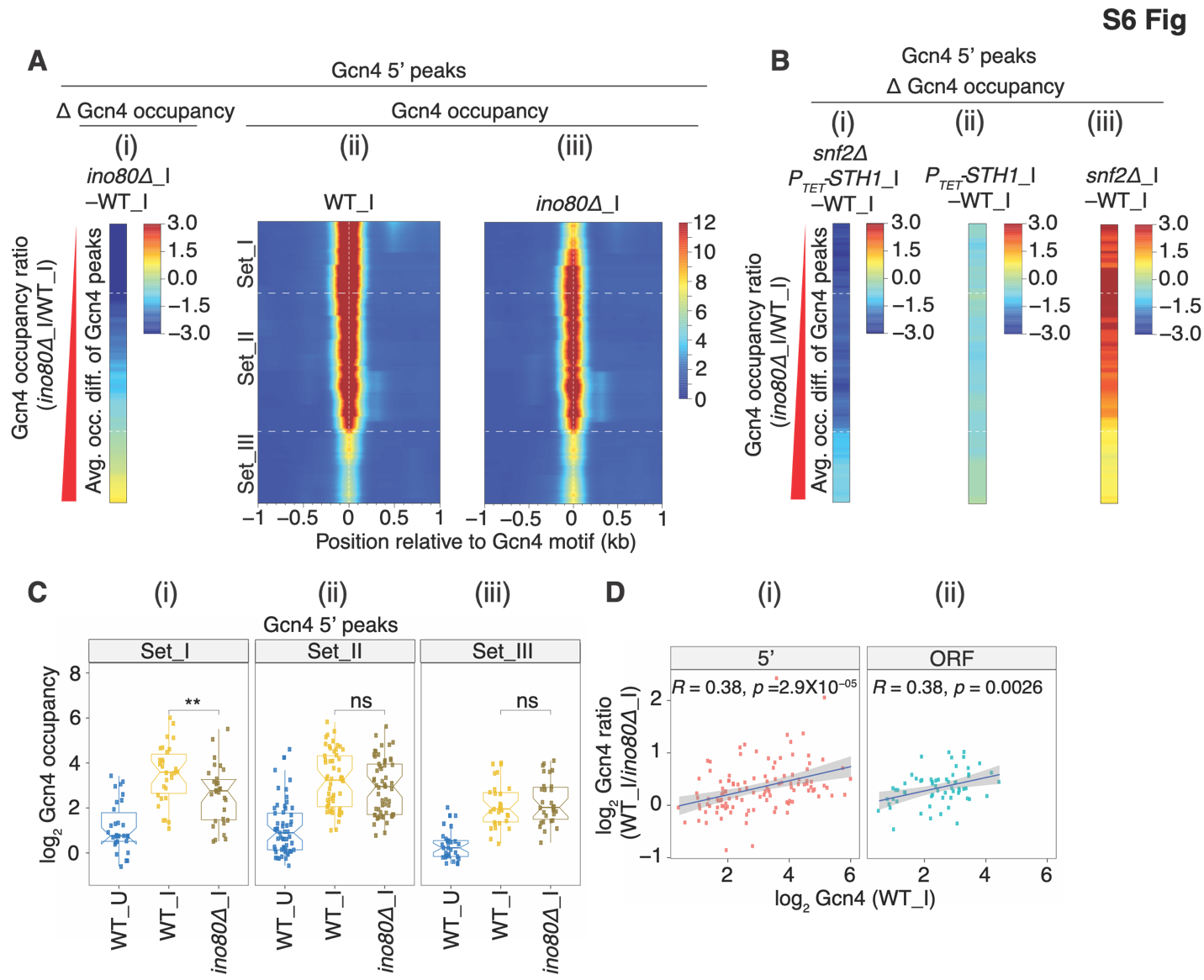


# S6 Fig. Identification of Gcn4 5’ sites with heightened Ino80C dependence for Gcn4 occupancy. (A) Heat map depicting differences in Gcn4 occupancies between *ino80Δ*_I and WT_I cells (i); Gcn4 occupancies surrounding the motifs of 5’ sites in (ii) WT_I or (iii) *ino80Δ*_I cells. Gcn4 5’ sites were sorted by increasing order of the ratio of Gcn4 occupancies in *ino80Δ*_I vs. WT_I cells, and the first (Set_I, n=30), middle two (Set_II, n=57) and fourth (Set_III, n=30) quartiles of fold-changes are depicted in A(ii). (B) Heat map depictions of differences in Gcn4 occupancies between (i) *snf2Δ P_TET_-STH1*_I and WT_I, (ii) *P_TET_-STH1_*I and WT_I*,* and (iii) *snf2*_I vs. WT_I cells in same order as in S6A Fig. (C) Notched box plots of log_2_ Gcn4 occupancy in WT_U, WT_I, and *ino80Δ*_I cells in 3 sets of Gcn4 5’ sites comprised of the (i) first (Set_I, n=30), (ii) middle two (Set_II, n=57) and (iii) last (Set_III, n=30) quartiles of the fold-changes in Gcn4 occupancy in *ino80Δ_I* vs. WT_I cells as defined in panel A(ii). *P* values for the significance of differences in medians calculated by the Mann-Whitney-Wilcoxon test are indicated. (D) Scatterplots of log_2_ ratios of Gcn4 occupancy changes in WT_I vs. *ino80Δ*_I cells plotted against log_2_ Gcn4 occupancies in WT_I cells for 5’ (i) and ORF (ii) Gcn4 sites*.* Pearson correlation coefficients (*R*) and associated *p* values are indicated.
