# Supplementary material for: Distinct functions of three chromatin remodelers in activator binding and preinitiation complex assembly
Source: PLoS Genet. 2022 Jul 6;18(7):e1010277. doi: 10.1371/journal.pgen.1010277 (PMC9292117; doi:10.1371/journal.pgen.1010277)
Supplement: S7 Fig — (A & B) Histograms depicting (A) log2 Rpb3 occupancies in the GCN4 CDS measured by Rpb3 ChIP-seq, indicating transcription levels; and (B) Gcn4 protein levels measured previously [12] by Western blot analysis in the indicated strains using Gcd6 signals analyzed in parallel as loading control. Band intensities for Gcn4 were normalized to those for Gcd6 in the same samples and the mean Gcn4/Gcd6 ratios determined from 3 biological replicates were plotted. Significance of differences in mean values was calculated with the student’s t test. (C & D) Scatterplots of log2 WT_I Gcn4 occupancies vs. motif FIMO scores [46] for (C) the 117 Gcn4 5’ sites and (D) the 62 Gcn4 ORF peaks, using the motif of highest score for peaks with multiple motifs. Pearson correlation coefficients (R) and associated p values are indicated. (E) Notched box plots of motif FIMO scores for the sets of Gcn4 5’ sites binned according to the fold-changes in occupancy in snf2Δ PTET-STH1_I vs. WT_I cells, as depicted in Fig 2B. (DOCX) [file pgen.1010277.s010.docx]

#
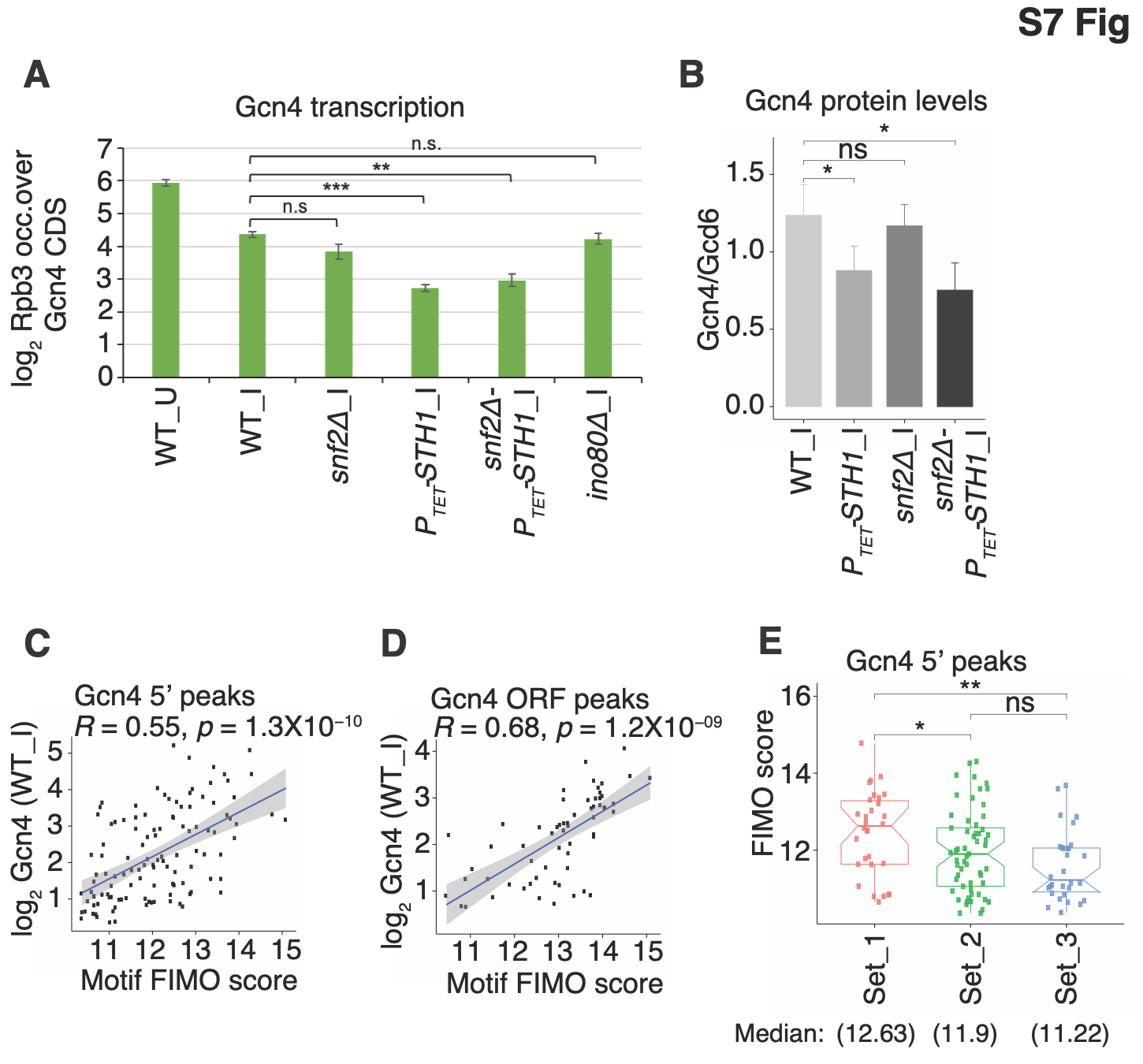


# S7 Fig. Supporting evidence that reduced Gcn4 binding in mutants depleted of RSC or Ino80C occurs preferentially at motifs of highest affinity or accessibility in chromatin in WT_I cells. (A & B) Histograms depicting (A) log_2_ Rpb3 occupancies in the *GCN4* CDS measured by Rpb3 ChIP-seq, indicating transcription levels; and (B) Gcn4 protein levels measured previously [1] by Western blot analysis in the indicated strains using Gcd6 signals analyzed in parallel as loading control. Band intensities for Gcn4 were normalized to those for Gcd6 in the same samples and the mean Gcn4/Gcd6 ratios determined from 3 biological replicates were plotted. Significance of differences in mean values was calculated with the student’s t test. (C & D) Scatterplots of log_2_ WT_I Gcn4 occupancies vs. motif FIMO scores [2] for (C) the 117 Gcn4 5’ sites and (D) the 62 Gcn4 ORF peaks, using the motif of highest score for peaks with multiple motifs. Pearson correlation coefficients (*R*) and associated *p* values are indicated. (E) Notched box plots of motif FIMO scores for the sets of Gcn4 5’ sites binned according to the fold-changes in occupancy in *snf2Δ P_TET_-STH1_*I vs. WT_I cells, as depicted in Fig 2B.

**REFERENCES**

1. Rawal Y, Chereji RV, Qiu H, Ananthakrishnan S, Govind CK, Clark DJ, et al. SWI/SNF and RSC cooperate to reposition and evict promoter nucleosomes at highly expressed genes in yeast. Genes Dev. 2018;32(9-10):695-710. doi: 10.1101/gad.312850.118. PubMed PMID: 29785963; PubMed Central PMCID: PMCPMC6004078.

2. Rawal Y, Chereji RV, Valabhoju V, Qiu H, Ocampo J, Clark DJ, et al. Gcn4 Binding in Coding Regions Can Activate Internal and Canonical 5' Promoters in Yeast. Mol Cell. 2018;70(2):297-311 e4. doi: 10.1016/j.molcel.2018.03.007. PubMed PMID: 29628310.
