# Supplementary material for: Distinct functions of three chromatin remodelers in activator binding and preinitiation complex assembly
Source: PLoS Genet. 2022 Jul 6;18(7):e1010277. doi: 10.1371/journal.pgen.1010277 (PMC9292117; doi:10.1371/journal.pgen.1010277)
Supplement: S8 Fig — The Gcn4 peak numbering assigned previously [46] is given at the top of each profile, and corrected Snf2-myc occupancies per nucleotide within ±100 bp windows surrounding the Gcn4 motifs are listed next to each peak. (DOCX) [file pgen.1010277.s011.docx]

#

# S8 Fig. Gene browser profiles of Gcn4 and corrected Snf2-myc occupancies from ChIP-seq analyses of sonicated chromatin for the indicated strains. The Gcn4 peak numbering assigned previously [1] is given at the top of each profile, and corrected Snf2-myc occupancies per nucleotide within ±100 bp windows surrounding the Gcn4 motifs are listed next to each peak.

**REFERENCE**

1. Rawal Y, Chereji RV, Valabhoju V, Qiu H, Ocampo J, Clark DJ, et al. Gcn4 Binding in Coding Regions Can Activate Internal and Canonical 5' Promoters in Yeast. Mol Cell. 2018;70(2):297-311 e4. doi: 10.1016/j.molcel.2018.03.007. PubMed PMID: 29628310.
