# Supplementary material for: Distinct functions of three chromatin remodelers in activator binding and preinitiation complex assembly
Source: PLoS Genet. 2022 Jul 6;18(7):e1010277. doi: 10.1371/journal.pgen.1010277 (PMC9292117; doi:10.1371/journal.pgen.1010277)
Supplement: S9 Fig — (A) (i)-(iii) Scatterplots of corrected occupancies of the indicated myc-tagged CR subunits in WT_I cells measured within ±100 bp windows surrounding the Gcn4 motifs of 5’ Gcn4 peaks. Pearson correlation coefficients (R) and associated p values are indicated. (B) Heat map depictions of corrected Ino80-myc occupancies at the 5’ Gcn4 peaks, sorted by decreasing Gcn4 occupancies in WT_I cells and plotted relative to the Gcn4 motifs, for (i) uninduced gcn4Δ cells, (ii) SM-treated gcn4Δ cells, (iii) uninduced WT cells, and (iv) SM-treated WT cells. Occupancies were calculated from ChIP-seq data of mildly sonicated chromatin from 2 or 3 biological replicates each of isogenic GCN4 or gcn4Δ strains, harboring INO80-myc or untagged INO80, under inducing or uninducing conditions, correcting the occupancies for INO80-myc cells for those measured for the untagged INO80 cells of the same GCN4 genotype and growth conditions. (C) Notched box plots of corrected Ino80-myc occupancies per nucleotide within ±100 bp windows surrounding the Gcn4 motifs of 5’ sites in uninduced (_U) or SM induced (_I) gcn4Δ or WT cells. (DOCX) [file pgen.1010277.s012.docx]

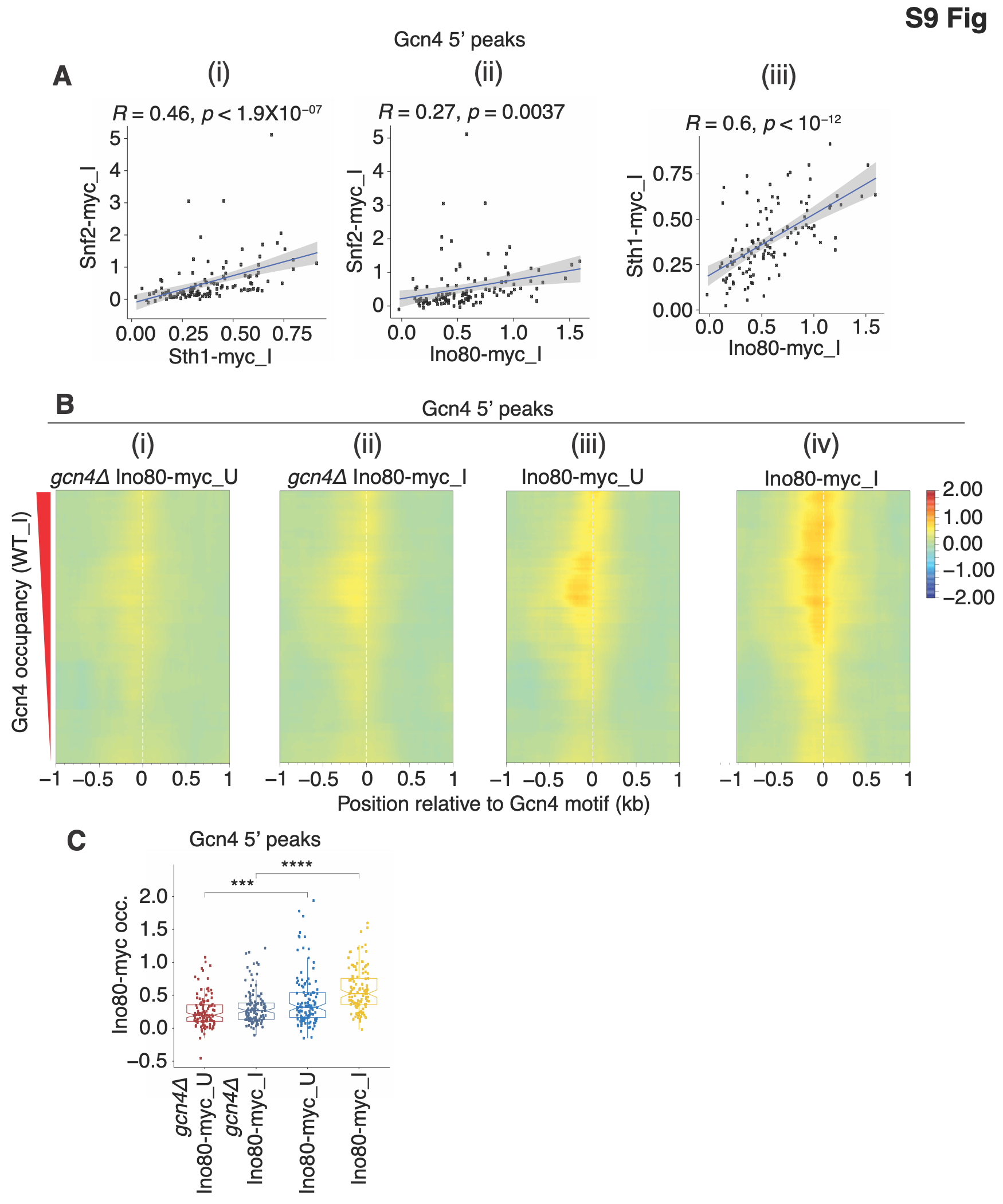


# S9 Fig. Supporting evidence for recruitment of the three CRs by Gcn4 to its 5’ sites. (A) (i)-(iii) Scatterplots of corrected occupancies of the indicated myc-tagged CR subunits in WT_I cells measured within ±100 bp windows surrounding the Gcn4 motifs of 5’ Gcn4 peaks*.* Pearson correlation coefficients (*R*) and associated *p* values are indicated. (B) Heat map depictions of corrected Ino80-myc occupancies at the 5’ Gcn4 peaks, sorted by decreasing Gcn4 occupancies in WT_I cells and plotted relative to the Gcn4 motifs, for (i) uninduced *gcn4Δ* cells, (ii) SM-treated *gcn4Δ* cells, (iii) uninduced WT cells, and (iv) SM-treated WT cells. Occupancies were calculated from ChIP-seq data of mildly sonicated chromatin from 2 or 3 biological replicates each of isogenic *GCN4* or *gcn4Δ* strains, harboring *INO80-myc* or untagged *INO80,* under inducing or uninducing conditions, correcting the occupancies for *INO80-myc* cells for those measured for the untagged *INO80* cells of the same *GCN4* genotype and growth conditions. (C) Notched box plots of corrected Ino80-myc occupancies per nucleotide within ±100 bp windows surrounding the Gcn4 motifs of 5’ sites in uninduced (_U) or SM induced (_I) *gcn4Δ* or WT cells.
