# Supplementary material for: Distinct functions of three chromatin remodelers in activator binding and preinitiation complex assembly
Source: PLoS Genet. 2022 Jul 6;18(7):e1010277. doi: 10.1371/journal.pgen.1010277 (PMC9292117; doi:10.1371/journal.pgen.1010277)
Supplement: S10 Fig — (A) Plots of H3 occupancies calculated from H3 MNase-ChIP-seq data at each base pair surrounding the Gcn4 motifs averaged over all 5’ sites for the indicated strains/conditions. (B) Notched box plots for the 3 sets of 5’ sites defined in Fig 2A and 2B depicting H3 occupancies per base pair in the ±100 bp windows surrounding the Gcn4 motifs. H3 occupancies were calculated from H3 MNase-ChIP-seq data from at least 3 biological replicates of WT_U, WT_I, or snf2Δ_I, PTET-STH1_I and snf2Δ PTET-STH1_I cells. (C-D) Sectored scatterplots of the log2 ratios of Gcn4 occupancies vs log2 ratios of H3 occupancies per base pair in the ±100 bp windows surrounding the Gcn4 motifs in snf2Δ PTET-STH1_I vs. WT_I cells (C) or snf2Δ_I vs. WT_I cells (D). The 3 sets of Gcn4 5’ sites defined in Fig 2B(ii) are color-coded as: Set_1, red rectangles; Set_2, green pluses; and Set_3, blue stars. (DOCX) [file pgen.1010277.s013.docx]

**S10 Fig. Supporting evidence for defective eviction of nucleosomes surrounding 5’ Gcn4 motifs in SWI/SNF and RSC mutants**. **(A)** Plots of H3 occupancies calculated from H3 MNase-ChIP-seq data at each base pair surrounding the Gcn4 motifs averaged over all 5’ sites for the indicated strains/conditions. **(B)** Notched box plots for the 3 sets of 5’ sites defined in Fig 2A-B depicting H3 occupancies per base pair in the ±100 bp windows surrounding the Gcn4 motifs. H3 occupancies were calculated from H3 MNase-ChIP-seq data from at least 3 biological replicates of WT_U, WT_I, or *snf2Δ*_I, *P_TET_-STH1_*I and *snf2Δ P_TET_-STH1_*I cells. **(C-D)** Sectored scatterplots of the log_2_ ratios of Gcn4 occupancies vs log_2_ ratios of H3 occupancies per base pair in the ±100 bp windows surrounding the Gcn4 motifs in *snf2Δ P_TET_-STH1_*I vs. WT_I cells (C) **or** *snf2Δ_*I vs. WT_I cells (**D**)**.** The 3 sets of Gcn4 5’ sites defined in Fig 2B(ii) are color-coded as: Set_1, red rectangles; Set_2, green pluses; and Set_3, blue stars.
