# Supplementary material for: Distinct functions of three chromatin remodelers in activator binding and preinitiation complex assembly
Source: PLoS Genet. 2022 Jul 6;18(7):e1010277. doi: 10.1371/journal.pgen.1010277 (PMC9292117; doi:10.1371/journal.pgen.1010277)
Supplement: S11 Fig — (A) (i)-(iii) Heat maps depicting differences between snf2Δ_I and WT_I cells for (i) Gcn4 occupancies measured as in Fig 2A(iii), (ii) H3 occupancies surrounding the Gcn4 motifs of 5’ sites from H3 ChIP-seq data, and (iii) Rpb3 occupancies averaged over the CDS of 5’ genes, for the Gcn4 5’ sites sorted by increasing order of fold-changes in Gcn4 occupancies in snf2Δ PTET-STH1_I vs. WT_I cells. (B) (i)-(iii) Same analyses shown in (A) except sorted by increasing order of fold-changes in Gcn4 occupancies in snf2Δ_I vs. WT_I cells. The locations of 5’ Gcn4 peaks in quartiles 1, 2–3 and 4 for changes in Gcn4 binding in snf2Δ_I vs. WT cells are indicated. (C) (i)-(iii) Same analyses shown in (A) except for PTET-STH1_I vs. WT_I data. (DOCX) [file pgen.1010277.s014.docx]

# S11 Fig. Defective eviction of nucleosomes associated with reduced Gcn4 occupancies at a subset of 5’ Gcn4 peaks in *snf2Δ*_I cells. (A) (i)-(iii) Heat maps depicting differences between *snf2Δ*_I and WT_I cells for (i) Gcn4 occupancies measured as in Fig 2A(iii), (ii) H3 occupancies surrounding the Gcn4 motifs of 5’ sites from H3 ChIP-seq data, and (iii) Rpb3 occupancies averaged over the CDS of 5’ genes, for the Gcn4 5’ sites sorted by increasing order of fold-changes in Gcn4 occupancies in *snf2Δ P_TET_-STH1*_I vs. WT_I cells. (B) (i)-(iii) Same analyses shown in (A) except sorted by increasing order of fold-changes in Gcn4 occupancies in *snf2Δ*_I vs. WT_I cells. The locations of 5’ Gcn4 peaks in quartiles 1, 2-3 and 4 for changes in Gcn4 binding in *snf2∆­*_I vs. WT cells are indicated. (C) (i)-(iii) Same analyses shown in (A) except for *P_TET_-STH1*_I vs. WT_I data.
