# Supplementary material for: Distinct functions of three chromatin remodelers in activator binding and preinitiation complex assembly
Source: PLoS Genet. 2022 Jul 6;18(7):e1010277. doi: 10.1371/journal.pgen.1010277 (PMC9292117; doi:10.1371/journal.pgen.1010277)
Supplement: S12 Fig — (A) (i)-(iii) Heat maps depicting differences between ino80Δ_I and WT_I cells for (i) Gcn4 occupancies measured as in S6A(i) Fig, (ii) H3 occupancies surrounding the Gcn4 motifs of 5’ sites from H3 ChIP-seq data, and (iii) Rpb3 occupancies averaged over the CDS of 5’ genes, for Gcn4 5’ sites sorted by increasing order of fold-changes in Gcn4 occupancies in ino80Δ_I vs. WT_I cells. (B-D) Notched box plots for the 3 sets of 5’ sites defined in S6A(ii) Fig, and indicated again in panel A(ii), depicting (B) H3 occupancies per base pair in the ±100 bp windows surrounding the Gcn4 motifs, (C) log2 Gcn4 occupancies taken from S6C Fig, and (D) log2 Rpb3 occupancies averaged over the CDS of genes with 5’ sites. H3 and Rpb3 occupancies were calculated from ChIP-seq data of sonicated chromatin from at least 3 biological replicates of WT_U, WT_I and ino80Δ_I cells. P values from Mann-Whitney-Wilcoxon tests are indicated. The heat map of H3 occupancy changes conferred by ino80Δ around the 5’ motifs ordered by the Gcn4 occupancy reductions in this mutant (panel A(i)) reveals that the 5’ sites with the strongest reductions in Gcn4 binding in ino80Δ cells located at the top of the map (Set_I) show the strongest increases in H3 occupancies centered around the Gcn4 motifs (panel A(ii)). Moreover, the decreases in median Gcn4 occupancy for individual 5’ sites conferred by ino80Δ are paralleled by increased median H3 occupancies for the Set_I group of 5’ sites; whereas the sites in Set_II and III show no significant changes in median H3 or Gcn4 occupancies in ino80Δ_I versus WT_I cells (panels 12B-C, Sets_I-III, col. 3 vs. 2). The subset of genes with 5’ sites that are most dependent on Ino80C for Gcn4 binding generally show the greatest reductions in Rpb3 occupancies (Set_I sites in panel A (iii) vs. (i)). Moreover, the Set_I genes, but not genes in Sets_II-III, show reduced median occupancies of both Gcn4 and Rpb3 in ino80Δ_I versus WT_I cells (panels C-D(i)-(iii), col. 3 vs. 2). As [file pgen.1010277.s015.docx]

# S12 Fig. Defective eviction of nucleosomes associated with reduced Gcn4 occupancies at a subset of 5’ Gcn4 peaks in *ino80Δ*_I cells. (A) (i)-(iii) Heat maps depicting differences between *ino80Δ*_I and WT_I cells for (i) Gcn4 occupancies measured as in S6A(i) Fig, (ii) H3 occupancies surrounding the Gcn4 motifs of 5’ sites from H3 ChIP-seq data, and (iii) Rpb3 occupancies averaged over the CDS of 5’ genes, for Gcn4 5’ sites sorted by increasing order of fold-changes in Gcn4 occupancies in *ino80Δ*_I vs. WT_I cells. (B-D) Notched box plots for the 3 sets of 5’ sites defined in S6A(ii) Fig, and indicated again in panel A(ii), depicting (B) H3 occupancies per base pair in the ±100 bp windows surrounding the Gcn4 motifs, (C) log_2_ Gcn4 occupancies taken from S6C Fig, and (D) log_2_ Rpb3 occupancies averaged over the CDS of genes with 5’ sites. H3 and Rpb3 occupancies were calculated from ChIP-seq data of sonicated chromatin from at least 3 biological replicates of WT_U, WT_I and *ino80Δ*_I cells. *P* values from Mann-Whitney-Wilcoxon tests are indicated. The heat map of H3 occupancy changes conferred by *ino80Δ* around the 5’ motifs ordered by the Gcn4 occupancy reductions in this mutant (panel A(i)) reveals that the 5’ sites with the strongest reductions in Gcn4 binding in *ino80Δ* cells located at the top of the map (Set_I) show the strongest increases in H3 occupancies centered around the Gcn4 motifs (panel A(ii)). Moreover, the decreases in median Gcn4 occupancy for individual 5’ sites conferred by *ino80Δ* are paralleled by increased median H3 occupancies for the Set_I group of 5’ sites; whereas the sites in Set_II and III show no significant changes in median H3 or Gcn4 occupancies in *ino80Δ*_I versus WT_I cells (panels 12B-C, Sets_I-III, col. 3 vs. 2). The subset of genes with 5’ sites that are most dependent on Ino80C for Gcn4 binding generally show the greatest reductions in Rpb3 occupancies (Set_I sites in panel A (iii) vs. (i)). Moreover, the Set_I genes, but not genes in Sets_II-III, show reduced median occupancies of both Gcn4 and Rpb3 in *ino80Δ*_I versus WT_I cells (panels C-D(i)-(iii), col. 3 vs. 2). As only the Set_I genes also exhibit increased median H3 occupancies in *ino80Δ*_I cells (panel B(i)-(iii), col. 3 vs. 2), it seems likely that a defect in Gcn4 binding (and subsequent impaired recruitment of other coactivators) in combination with loss of Ino80C-mediated promoter nucleosome eviction produces the reduced transcription of Set_I genes conferred by *ino80Δ*. (E) Gene browser profiles of Gcn4 and H3 occupancies from ChIP-seq analyses of sonicated chromatin for the indicated strains, as described in S4 Fig.
