# Supplementary material for: Distinct functions of three chromatin remodelers in activator binding and preinitiation complex assembly
Source: PLoS Genet. 2022 Jul 6;18(7):e1010277. doi: 10.1371/journal.pgen.1010277 (PMC9292117; doi:10.1371/journal.pgen.1010277)
Supplement: S13 Fig — (A-B) Averaged TBP occupancies surrounding the Gcn4 motifs in ORF peaks from ChIP-seq data of sonicated chromatin using anti-TBP antibodies from at least 2 biological replicates for the indicated mutant and WT strains. (C) Notched box plots of factor occupancies in the 83 5’ gene promoters for the two bins defined in Fig 5C for (i) log2 TBP within ±100 bp of TBP peak summits and (ii) H3 within ±100 bp of the TBP peak summits. P values from Mann-Whitney-Wilcoxon tests are indicated. (D-E) Gene browser profiles of TBP, Rpb3, and H3 occupancies from ChIP-seq analyses of sonicated chromatin for the indicated strains. The TBP occupancies per nucleotide over the TBP peaks and Rpb3 occupancies per nucleotide over the CDS are listed next to the relevant peaks or CDSs. The locations of the Gcn4 motifs and TBP summits are indicated with vertical hash marks, and the positions of -1 and +1 nucleosomes with dashes, at the bottom of each profile. (DOCX) [file pgen.1010277.s016.docx]

# S13 Fig. Decreased TBP recruitment at 5’ and ORF genes in *ino80Δ*_I cells. (A-B) Averaged TBP occupancies surrounding the Gcn4 motifs in ORF peaks from ChIP-seq data of sonicated chromatin using anti-TBP antibodies from at least 2 biological replicates for the indicated mutant and WT strains. (C) Notched box plots of factor occupancies in the 83 5’ gene promoters for the two bins defined in Fig 5C for (i) log_2_ TBP within ±100 bp of TBP peak summits and (ii) H3 within ±100 bp of the TBP peak summits. *P* values from Mann-Whitney-Wilcoxon tests are indicated. (D-E) Gene browser profiles of TBP, Rpb3, and H3 occupancies from ChIP-seq analyses of sonicated chromatin for the indicated strains. The TBP occupancies per nucleotide over the TBP peaks and Rpb3 occupancies per nucleotide over the CDS are listed next to the relevant peaks or CDSs. The locations of the Gcn4 motifs and TBP summits are indicated with vertical hash marks, and the positions of -1 and +1 nucleosomes with dashes, at the bottom of each profile.
