# Supplementary material for: Distinct functions of three chromatin remodelers in activator binding and preinitiation complex assembly
Source: PLoS Genet. 2022 Jul 6;18(7):e1010277. doi: 10.1371/journal.pgen.1010277 (PMC9292117; doi:10.1371/journal.pgen.1010277)
Supplement: S14 Fig — The TBP occupancies per nucleotide over the TBP peaks and Rpb3 occupancies per nucleotide over the CDS are listed next to the relevant peaks or CDSs. The locations of the Gcn4 motifs and TBP summits are indicated with vertical hash marks, and the positions of -1 and +1 nucleosomes with dashes, at the bottom of each profile. (DOCX) [file pgen.1010277.s017.docx]

**S14 Fig. Gene browser profiles of TBP, Rpb3, and H3 occupancies from ChIP-seq analyses of sonicated chromatin for the indicated strains.** The TBP occupancies per nucleotide over the TBP peaks and Rpb3 occupancies per nucleotide over the CDS are listed next to the relevant peaks or CDSs. The locations of the Gcn4 motifs and TBP summits are indicated with vertical hash marks, and the positions of -1 and +1 nucleosomes with dashes, at the bottom of each profile.
