# Supplementary material for: Distinct functions of three chromatin remodelers in activator binding and preinitiation complex assembly
Source: PLoS Genet. 2022 Jul 6;18(7):e1010277. doi: 10.1371/journal.pgen.1010277 (PMC9292117; doi:10.1371/journal.pgen.1010277)
Supplement: S15 Fig — Changes in TBP occupancies surrounding the TSSs in the indicated mutants versus WT under non-starvation conditions are plotted for the coactivator-redundant and TFIID-dependent genes defined by Donczew et al. (2020), along with the corresponding changes for the RPGs. (DOCX) [file pgen.1010277.s018.docx]

**S15 Fig. Coactivator-redundant genes have a greater requirement than TFIID-dependent genes for RSC and Ino80C for TBP recruitment.** Changes in TBP occupancies surrounding the TSSs in the indicated mutants versus WT under non-starvation conditions are plotted for the coactivator-redundant and TFIID-dependent genes defined by Donczew et al. (2020), along with the corresponding changes for the RPGs.

**Reference:**

Donczew R, Warfield L, Pacheco D, Erijman A, Hahn S. Two roles for the yeast transcription coactivator SAGA and a set of genes redundantly regulated by TFIID and SAGA.

Elife. 2020; 9:e50109. doi: 10.7554/eLife.50109. PubMed PMID: 31913117. PubMed Central PMCID: PMC6977968.
